# Biochemical and Molecular Effects Induced by Triacontanol in Acquired Tolerance of Rice to Drought Stress

**DOI:** 10.3390/genes12081119

**Published:** 2021-07-23

**Authors:** Basmah M. Alharbi, Awatif Mahfouz Abdulmajeed, Heba Hassan

**Affiliations:** 1 Biology Department, Faculty of Science, University of Tabuk, Tabuk 71421, Saudi Arabia; b.alharbi@ut.edu.sa; 2 Biology Department, Faculty of Science, University of Tabuk, Umluj 46429, Saudi Arabia; awabdulmajeed@ut.edu.sa; 3 Botany Department, Faculty of Science, Ain Shams University, Cairo 11566, Egypt

**Keywords:** triacontanol, drought, rice, aquaporins, *PIP1,1*, *PIP1,2*, *PIP2,4* and *PIP2,5* genes

## Abstract

To assess the effect of triacontanol (TRIA) on rice plants grown under normal or drought conditions, rice seeds were presoaked in TRIA (35 ppm) for two hours. After 20 days of sowing, rice seedlings developed from TRIA-treated or untreated seeds were subjected to drought stress. After 10 days of plant exposure to drought stress, data of major growth attributes and the content of photosynthetic pigments were recorded. Moreover, the effect of drought stress on stomatal conductance and the photochemical efficiency of PSII (Fv/Fm) were followed. The data obtained indicated that the species of rice (*Oryza sativa* L.) cultivar Giza 177 under investigation was sensitive to drought stress where there were significant decreases in the fresh and dry weights of shoots and roots and in stomatal conductance, as well as in the content of chlorophyll a, chlorophyll b, and carotenoids. Seed priming with TRIA enhanced both growth and acquired plant tolerance to drought stress. Thus, TRIA via the enhancement of stomatal conductance through the regulation of stomatal closure, the rate of water loss, ABA metabolism, the accumulation of osmolytes, and the regulation of aquaporins genes improved the water status of plants grown under water scarcity. Moreover, TRIA via increasing the content of free amino acids and sugars under drought stress may increase the chance of plant tissues to retain more water under scarcity conditions.

## 1. Introduction

Drought is one of the major environmental constrictions limiting plant development and productivity [1]. Drought menaces about 70% of arable land worldwide. Consequently, the major crops will exhibit over 65% reduction in their yield by 2050 because of drought all over the world [2,3]. Rice is one of the major staple food crops for most of world population, and belongs to semi-aquatic plants, so it requires a high soil moisture level [4]. Thus, rice plants are susceptible to water scarcity, which induces a variety of morphological, molecular, and physiological changes [5]. It was reported that major growth attributes of important crops are severely affected by drought stress [6]. It was also reported that drought stress disturbs the leaf water potential, transpiration rate, and stomatal conductance [7]. Moreover, drought induces oxidative stress that destroys various macromolecules as proteins, lipids, and nucleic acids concomitant with cell membranes damage [8]. It was recorded that seedling growth, dry weight, and vegetative growth were reduced under drought stress in various important crops including pea (*Pisum sativum* L.), alfalfa (*Medicago sativa* L.), and rice (*Oryza sativa* L.) [6,9,10].

Plants can tolerate drought stress by developing different structural, biochemical, and molecular strategies including accumulation of certain osmolytes and proteins [11]. Indeed, drought stress accelerates abscisic acid (ABA) biosynthesis, which plays a crucial role in stomatal conductance [12,13]. In this connection, the accumulation of root ABA under drought stress was reported in many plants such as rice, beans, and potato [14,15,16].

Furthermore, plants can cope with drought stress by stimulating the expression of various genes as genes of some protective proteins, water channel proteins (aquaporins), enzymes catalyzing osmolyte biosynthesis, proteases, and detoxification enzymes. Similarly, genes encode various proteins such as kinases, transcription factors, phosphatases, and enzymes that regulate certain pathways including ABA biosynthesis and phospholipid metabolism were also regulated under drought condition [11,17]. Aquaporins (AQPs) are a class of intrinsic proteins that play an important role in regulating the transmembrane transport of water [18,19] and small molecules like glycerol, urea, and CO_2_ [20,21]. Many types of AQPs are known in plants for their importance in stabilizing cell membrane homeostasis and keeping movement of water through the plant body under drought conditions. Plasma membrane intrinsic proteins (PIP) belong to one of the subfamilies of AQPs, and PIPs are further subdivided into two phylogenetic subgroups: PIP1s and PIP2s [22].

Triacontanol (TRIA), the material used as a seed primer in the present work, is a saturated primary alcohol classified as a plant growth regulator (PGR) that stimulates many physiological and biochemical processes in crop plants [23,24]. Triacontanol at relatively low concentrations enhances the growth of most crops such as rice (*O. sativa* L.) and maize (*Zea mays* L.) [25,26]. Currently, TRIA has been used to improve plant tolerance against abiotic stresses such as chilling, drought, and heavy metal and salt stresses [25,27,28]. Notably, under abiotic stresses, exogenously applied TRIA stimulates growth, increases the content of photosynthetic pigments, and increases compatible osmolyte accumulation [29,30]. Additionally, it enhances enzymatic and non-enzymatic antioxidant defense systems [27,30,31,32,33]. TRIA can also mitigate stress hazards via the regulation of the expression of some genes [28,32]. The present work investigates the efficiency of TRIA in enhancing drought tolerance of rice plants.

## 2. Materials and Methods

### 2.1. Materials and Growth Conditions

Grains of rice (*O. sativa* L.) cultivar Giza 177 were obtained from the Agriculture Research Center, Rice Research Institute in Giza, Egypt. Triacontanol (TRIA) was obtained from Sigma-Aldrich (Lot 637238, St. Louis, MO, USA). This research was conducted at Faculty of Science, Ain Shams University, Egypt using two controlled growth chambers, model V3-DM, Vision scientific company, Korea. The grains were surface sterilized by immersion in 1% (*w/v*) sodium hypochlorite solution for 5 min, then washed three times with sterile distilled water prior to an experimental procedure to prevent fungal contamination.

### 2.2. Imposition of Treatments

The sterilized grains were divided into two groups, which were soaked either in water or TRIA (35 ppm) for 2 h. The experiment was conducted in a naturally lit greenhouse (day/night temperatures about 27/32 ± 2 °C and a 14 h photoperiod) of the Botany Department, Faculty of Science, Ain Shams University. This experiment was carried out in a complete randomized design with three replicates. The sterilized rice grains of the two groups were sown in plastic pots (25 × 25 cm^2^) filled with homogenous soil (50 pots for each group). The physical and chemical analysis of soil are given in Table 1.

The irrigation of all pots was carried out using the same volume of water based on the maximum water-holding capacity of the soil used in the present work. After 15 days of sowing, thinning was done so that 10 uniform seedlings were left in each pot. After 20 days of sowing, drought stress was imposed on half of each group by withholding irrigation. After 10 days of rice seedling exposure to drought stress, the experiment was terminated as severe growth retardation was observed, compared with the control or with seedlings developed from pre-soaked grains in TRIA. Both shoots and roots were collected directly frozen in liquid nitrogen and then stored at −80 °C for biochemical analyses.

### 2.3. Methods

#### 2.3.1. Measurement of Number of Stomata

Direct microscopic measurements of number of stomata were carried out following the method described by [34]. Leaf epidermal strips were obtained from a fully expanded leaf and immediately immersed in absolute alcohol for fixation and preservation. The epidermal strip was prepared on a slide and then covered with a cover slip. The total number of stomata as well as number of open stomata per µm^2^ on the upper and lower epidermis were counted using an eye-piece graticule, which is calibrated by using stage micrometer scale.

#### 2.3.2. Chlorophyll Fluorescence Measurements

The chlorophyll a fluorescence measurement was assessed in leaves in the morning hours. The intact flag leaves were dark adapted for 30 min using light-withholding clips. Leaf chlorophyll fluorescence was measured simultaneously using a pulse amplitude modulation portable fluorometer (Handy PEA, Hansatech, Norfolk, UK). After the adaptation of leaves to darkness, a single, strong, 1 s light pulse (3500 μmol m^2^ s) was applied. Three replicates were used for each treatment. The fast fluorescence kinetics (F_0_ to Fm) value was recorded during 10 μs to 1 s [35]. The maximum quantum efficiency of PSII photochemistry (Fv/Fm) was calculated according to the equation:

Fv/Fm = (Fm − F_0_)/Fm

where F_0_ means fluorescence intensity at 50 μs, Fm represents maximal fluorescence intensity, and Fv represents variable fluorescence.

#### 2.3.3. Measurement of Photosynthetic Pigments

The photosynthetic pigments chlorophyll (Chl) a, Chl b, and carotenoids were extracted and determined according to the method of [36]. Fresh leaves (1 g) were homogenized in 85% aqueous acetone for 5 min. Then, the homogenate was centrifuged, and the supernatant was made up to 100 mL with 85% acetone. The extinction was measured against a blank of pure 85% aqueous acetone at three different wave lengths (452.5, 644, and 663 nm) by using spectrophotometer (Spectronic 601, Milton Roy Company, Ivyland, PA, USA) 

#### 2.3.4. Measurement of Total Soluble Sugars

Total soluble sugars were analyzed by reacting 0.1 mL of the ethanolic extract with 3 mL of freshly prepared anthrone reagent (150 mg anthrone + 100 mL 72% H_2_SO_4_) in boiling water bath for 10 min. After cooling, the absorbance was measured at 620 nm by using a spectrophotometer [37].

#### 2.3.5. Measurement of Free Amino Acids

Free amino acids were determined according to [38] by grinding the plant tissue (0.5 g) in water; then, 0.1 mL of the water extract was added to 1.5 mL (ethanol/acetone) of a 1:1 (*v*/*v*) mixture of 0.1 mL phosphate buffer (pH 6.5) and 2 mL ninhydrin reagent (0.5% in n-butanol). Then, the mixture was placed in a boiling water bath for 20 min, and then cooled immediately in ice water, and methanol was added to 10 mL. The absorbance was measured directly at 580 nm by using spectrophotometer.

#### 2.3.6. Determination of Free Proline

The total free proline was assessed by the method described by [39] using ninhydrin reagent. The plant tissue (0.5 g) was grinded in 6 mL of 3% (*w*/*v*) sulfosalicylic acid solution. Then, the filtrate (2 mL) was reacted with 2 mL ninhydrin reagent and 2 mL glacial acetic acid, and the mixture was kept in boiling water bath for 1 h. Then, the mixture was cooled in ice and was separated using a separating funnel. The absorbance of the upper phase was read at 520 nm by using spectrophotometer.

#### 2.3.7. Determination of Electrolyte Leakage (EL)

The stress injury was measured by electrolyte leakage as described by [40]. Leaf samples (0.5 g) were incubated with 20 mL of deionized water for 24 h at 25 °C. Then, the electrical conductivity of the solution (L_1_) was quantified using a conductivity meter (HI 8733, Hanna Instruments, Woonsocket, RI, USA). Samples were then autoclaved at 120 °C for 20 min and then the final conductivity (L_2_) was assessed after equilibration at 25 °C. The EL was determined according to the following equation:

EL% = (L_1_/L_2_) × 100

#### 2.3.8. Lipid Peroxidation

Lipid peroxidation was determined by measuring the amount of malondialdehyde (MDA) produced by the thiobarbituric acid reaction as described by [41]. The plant tissue (0.5 g) was grinded in water then the crude extract was mixed with the same volume of a 0.5% (*w*/*v*) thiobarbituric acid solution containing 20% (*w*/*v*) trichloroacetic acid. The mixture was heated at 95 °C for 30 min and then quickly cooled in an ice-bath. The mixture was centrifuged at 3000× *g* for 5 min and the absorbance of the supernatant was measured at 532 and 600 nm by using a spectrophotometer.

#### 2.3.9. Relative Water Content (RWC)

The relative water content was measured following the method described by [42]. Leaf discs from the fully expanded and uniform leaves were taken. The fresh mass (FM) of leaf discs was measured, and then samples were placed in a Petri dish with distilled water for 4 h. The water saturated mass (WSM) was then measured, and the leaf samples were placed in an oven at 80 °C for 48 h to determine the dry mass (DM). Leaf RWC was calculated as:

RWC [%] = [(FM − DM)/(WSM − DM] × 100

#### 2.3.10. Extraction, Separation, and Determination of Abscisic Acid (ABA)

The method of hormones extraction was essentially similar to that adopted by [43]. The frozen tissue was homogenized in cold 85% ethanol by an electric automixer and then extracted by an electric stirrer with 85% ethanol at about 0 °C. The solvent was changed three times. After filtration, the three extracts were combined together and concentrated under a vacuum at 20–25 °C to a few mL. The concentrated aqueous phase was adjusted to pH 8.8 by using 1% NaOH. The alkaline aqueous phase was shaken three times with equal quantities of ethyl acetate using a separating funnel. The combined ethyl acetate fraction was evaporated to dryness and held for further purification. The aqueous fraction was acidified to pH 2.8 with 1% HCI and shaken three times with equal volumes of ethyl acetate. The remaining aqueous phase was discarded. The combined acidic ethyl acetate phase was reduced to a certain volume to determine the abscisic acid (ABA) by using gas chromatography (GC). The dried basic ethyl acetate fraction was dissolved in 80% methanol.

#### 2.3.11. Quantitative Real-Time PCR (qRT-PCR) Analysis

The total RNA was extracted from rice tissue (100 mg) of all treatments with 30% PEG6000 using the RNeasy Plant Mini Kit (Qiagen, Amsterdam, The Netherlands). The total RNA (1 µg) from each sample was transformed into cDNA by the reverse transcription using the c.DNA Kit (TaKaRa) according to the manufacturer’s instructions. The qRT-PCR was conducted on an ABI 7500 system (Applied Biosystems, New York, NY, USA) using a TransStart™ Green qRT-PCR Super Mix Kit (TransGen, Beijing, China). OsActin rRNA was used as a reference gene to standardize the relative transcriptional abundance and to minimize different copy numbers of cDNA templates [44]. All data were calculated from three replicates based on the 2^−∆∆Ct^ method [45]. The primers of the *PIP1,1*, *PIP1,2*, *PIP2,4,* and *PIP2,5* genes (Table 2) used in the qRT-PCR excluded the highly conserved protein domain and had high efficiency and specificity.

### 2.4. Statistical Analysis

The experimental data presented in this work were statistically analyzed by the one-way analysis of variance (ANOVA) using SPSS v20.0 (SPSS Inc., Chicago, IL, USA) analyzing software. Statistical significances of the means were compared with Duncan’s test at *p* ≤ 0.05 levels and the standard error (SE) of the means are shown in tables and figures as mean ± SE, with the number of degrees of freedom (*n*) = 3.

## 3. Results

**Figure 1:**
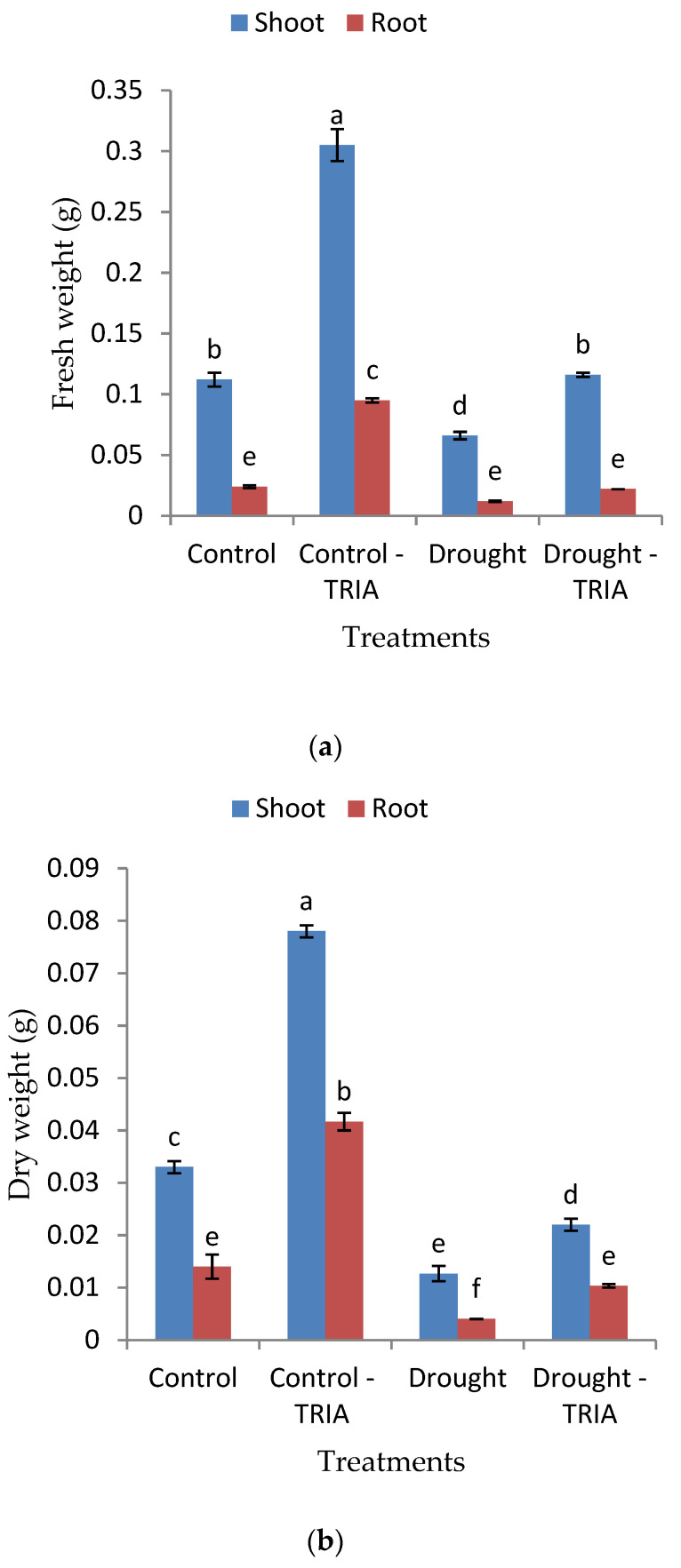

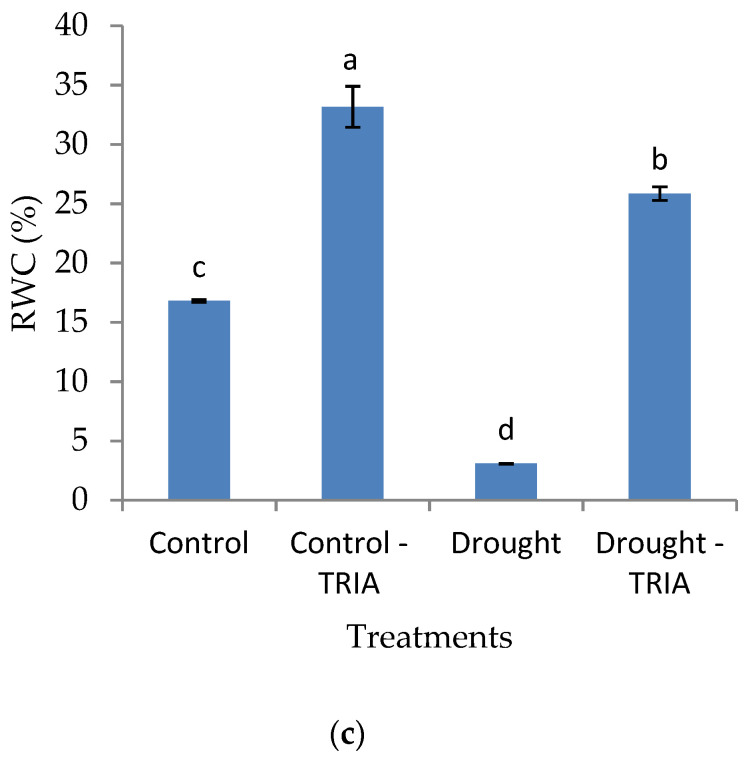
Effect of TRIA (35 ppm) treatment on (**a**) fresh weight, (**b**) dry weight, and (**c**) relative water content of drought-stressed rice seedlings. Each value is the mean of three replicates ± SE. Columns with different letters are significantly different at *p* ≤ 0.05.

TRIA treatment of unstressed rice seedlings led to significant increases in the fresh and dry weights of shoots and roots as compared with the unstressed control (Figure 1a,b). Meanwhile, the imposition of drought stress induced a significant decrease in the fresh and dry weights of both shoots and roots as compared with the unstressed control, while stressed plants treated with TRIA showed an increase in the fresh and dry weights as compared with the stressed control (Figure 1a,b).

Moreover, the results obtained showed that the relative water content was decreased in the leaves of rice seedlings exposed to drought. On the other hand, the pretreatment with TRIA significantly increased the relative water content of stressed leaves (Figure 1c).

Likewise, drought stress induced a significant decrease in the leaf content of Chl a, Chl b, and carotenoids (Table 3). Notably, TRIA pretreatment induced a significant increase in Chl a, Chl b, and the contents of leaves of drought-stressed seedlings, compared with the untreated stressed controls (Table 3). TRIA increased the photosynthetic pigments concomitant with increments in the Fv/Fm values of the leaves of stressed rice seedlings (Table 3).

**Figure 2:**
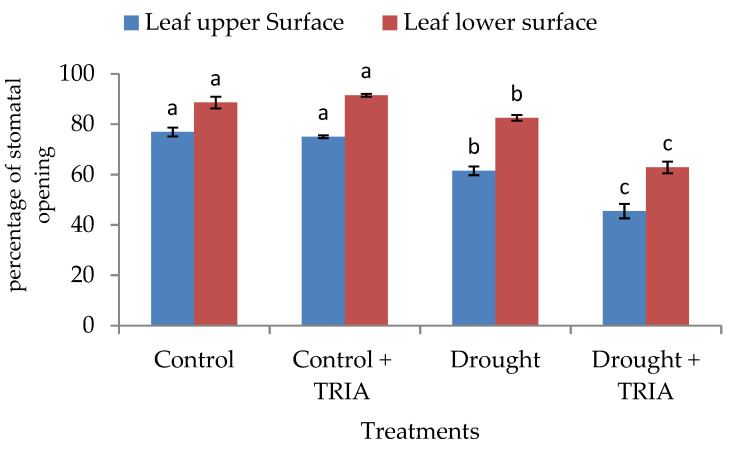
Effect of TRIA (35 ppm) treatment on percentage of stomatal openings on both the upper and lower surface of leaves of drought-stressed rice. Each value is the mean of three replicates ± SE. Columns with different letters are significantly different at *p* ≤ 0.05.

Moreover, in this study, drought obviously induced a significant decrease in percentage of open stomata (Figure 2) as compared with the unstressed control. The pretreatment of rice with TRIA significantly reduced the percentage of stomatal openings on both the upper and lower surface of rice leaves by 26% and 23.9%, respectively, as compared with the stressed control.

**Figure 3:**
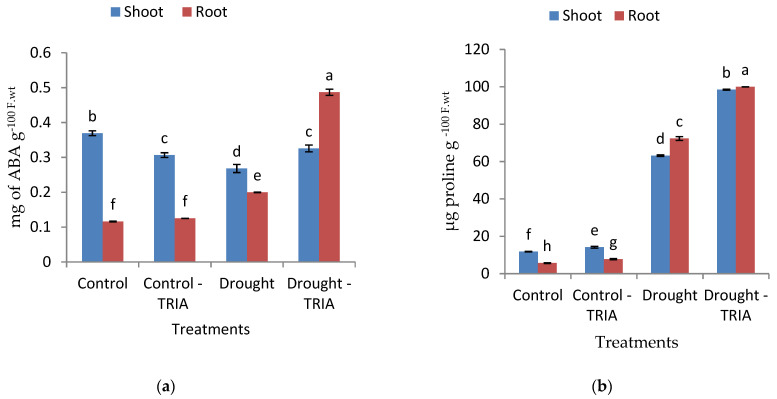

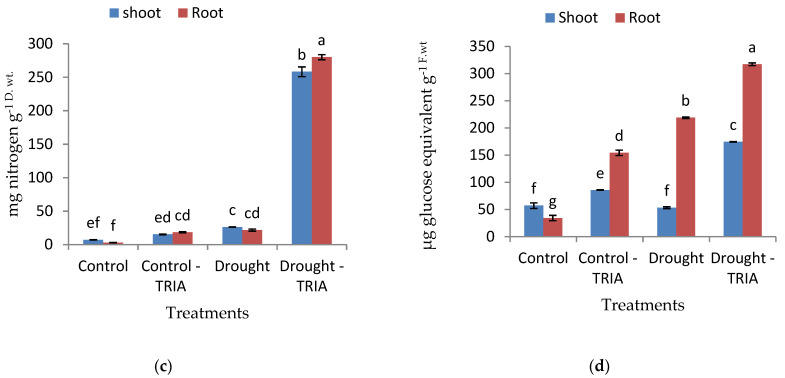
Effects of TRIA (35 ppm) treatment on (**a**) abscisic acid, (**b**) free proline, (**c**) free amino acids, and (**d**) total soluble sugars of drought-stressed rice seedlings. Each value is the mean of three replicates ± SE. Columns with different letters are significantly different at *p* ≤ 0.05.

The current data also revealed that exposure to drought stress significantly increased and decreased the ABA content of the roots and shoots, respectively (Figure 3a). In addition, proline and amino acids showed a significant increase in both the shoots and roots of drought-stressed seedlings as compared with the unstressed control (Figure 3b,c).

Meanwhile, the total soluble sugars recorded a non-significant decrease and a significant increase in the shoots and roots of drought-stressed seedlings, respectively (Figure 3d). On the other hand, TRIA treatment of drought-stressed rice seedlings led to a significant increase in ABA, proline, amino acids, and total soluble sugar contents in both the shoots and roots (Figure 3a–d).

**Figure 4:**
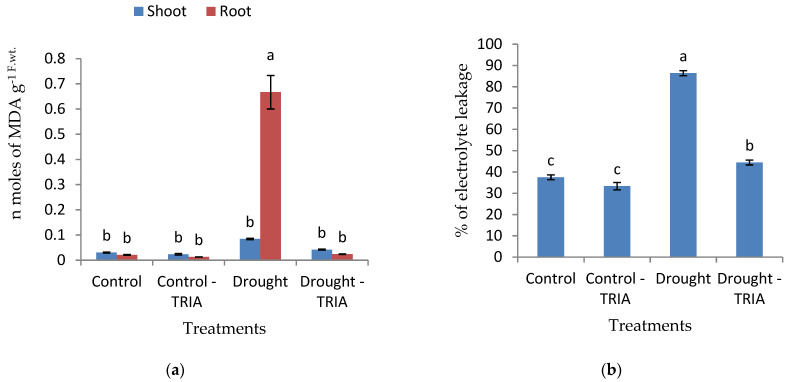
Effects of TRIA (35 ppm) treatment on (**a**) malondialdehyde (MDA) content and (**b**) the percentage of electrolytes leakage (EL) of drought-stressed rice seedlings. Each value is the mean of three replicates ± SE. Columns with different letters are significantly different at *p* ≤ 0.05.

Furthermore, drought stress induced a significant increase in the lipid peroxidation product, concomitant with a significant increase in the electrolyte leakage value (Figure 4a,b), respectively, as compared with those of the unstressed plants. The pretreatment with TRIA significantly reduced the lipid peroxidation product and electrolyte leakage of drought-stressed rice seedlings (Figure 4a,b).

**Figure 5:**
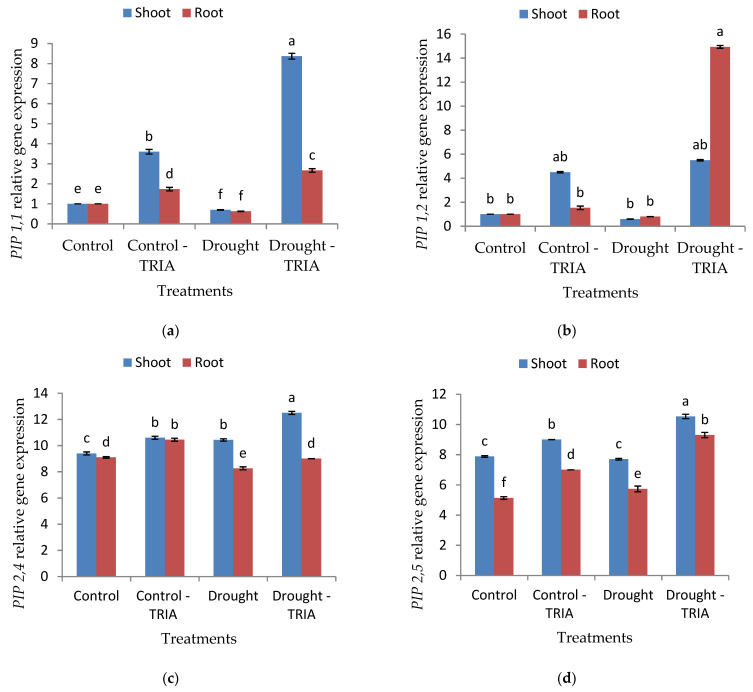
Effects of TRIA (35 ppm) treatment on mRNA expression of plasma membrane intrinsic protein (**a**) PIP1,1, (**b**) PIP1,2, (**c**) PIP2,4, and (**d**) PIP2,5 of drought-stressed rice seedlings. Each value is the mean of three replicates ± SE. Columns with different letters are significantly different at *p* ≤ 0.05.

Notably, the RT-PCR analysis showed that drought downregulated *PIP1,1*, *PIP1,2, PIP2,4,* and *PIP2,5* expressions (Figure 5a–d). Meanwhile, TRIA pretreatment stimulated the overexpression of *PIP1,1*, *PIP1,2*, *PIP2,4*, and *PIP2,5* of drought-stressed rice shoots and roots (Figure 5a–d) as compared with the stressed, untreated plants. The maximum expressions of *PIP1,1*, *PIP2,4,* and *PIP2,5* were observed in TRIA-primed stressed leaves; however, the greatest expression of *PIP1,2,* was assayed in TRIA-primed stressed roots.

## 4. Discussion

Drought stress adversely affects plant growth and development. On the other hand, the ability to survive the drought state is a result of adaptations that prevent or decrease cellular destruction that occurs with the scarcity of water. The results presented in Figure 1a,b showed a significant decrease in both the fresh and dry weighs of shoots and roots of rice exposed to drought conditions. Such an effect was concomitant with a significant decrease in the relative water content (RWC) of rice leaves (Figure 1c), which may reduce the cell turgor pressure and, thus, cause growth retardation [46,47]. The drop in RWC of drought-stressed seedlings may be attributed to a decline in water uptake by roots, which was recorded by [48]. Moreover, drought adversely affects the photosynthetic process, as it caused a decline in the photosynthetic pigments content in our study (Table 3). This effect may be attributed to destruction or photooxidation of chlorophyll and/or inhibition of Chl synthesis, or to an increase in the activity of chlorophyllase [48,49].

Triacontanol (TRIA) is a promising plant growth regulator, as it plays an active role in the upregulation of major physiological activities required in different stages of plant growth [50,51]. In the present study, the priming of rice grains with TRIA (35 ppm) increased the fresh and dry weights of the shoots and roots of rice grown under well-watered conditions (Figure 1a,b) compared with plants exposed to drought stress. Similarly, TRIA enhanced the growth of several crops like ginger [23], tomato [52], rice [53], and viviparous [54]. The significant promoting effects of TRIA on the fresh and dry weights of TRIA-treated rice seedlings concomitant with increases in the chlorophyll a, chlorophyll b, and carotenoid contents of leaves (Table 3) demonstrated the motivating effect of TRIA on the photosynthetic efficiency, which, sequentially, improved the assimilation rate and the accumulation of photosynthates. In this regard, it has been reported by many researchers that TRIA stimulated photosynthesis in several crops such as *Papaver somniferum* L. [55], *Vigna radiata* L. [56], and *Lablab purpureus* L. [57]. Given this connection, it was reported that TRIA may enhance the photosynthetic process via increasing the rate of chlorophyll synthesis, as well as the number and size of chloroplasts [58,59,60]. Moreover, Fv/Fm values were markedly increased in TRIA-treated rice seedlings (Table 3), and thereby, may contribute to improving the photosynthetic efficiency of PSII and to lessening the degree of photoinhibition [59,61]. In addition, TRIA may increase photosynthesis via enhancing the activity of the Rubisco enzyme, photosynthetic pigments, and the upregulation of many photosynthetic genes [23,62].

Now it has been well documented that TRIA functions as a signaling molecule and accelerates plant tolerance against various abiotic stresses [25,63]. TRIA clearly improved seedling growth, as measured by both the fresh and dry weights of rice seedlings exposed to drought stress as compared with the stressed control ones (Figure 1a,b). Similar results were obtained by [64,65] on drought-stressed *V. radiata* L and rice seedlings, respectively. The increments in the previous parameters may be attributed to the significant increase obtained in the RWC (Figure 1c) and the significant decrease obtained in the percentage of stomatal opening (Figure 2), which may have played a crucial role in controlling water loss. The stomatal closure is an important strategy to avoid water loss and help plants tolerate drought conditions [7,66]. TRIA as a constituent of wax in the cuticle of plants [67] might have a role in controlling the rate of water loss and might have counteracted the drought-induced disturbance in the leaf water potential. Moreover, TRIA treatment increased the abscisic acid content (ABA) of the shoots and roots of drought-stressed rice seedlings as compared with the control (Figure 3a). ABA is among the hormones that affect the water status of plants via regulation of the stomatal function [68,69].

In addition, the results obtained suggested that TRIA priming helped plants to improve the water status under drought stress through osmotic adjustment, as attained by the accumulation of some osmolytes, including proline, free amino acids, and total soluble sugars (Figure 3b–d). The accumulation of proline, total soluble sugars, and free amino acids helps in reducing the cell osmotic potential, thereby diminishing water loss under water scarcity [70]. In the present investigation, the priming of rice grains with TRIA stimulated the accumulation of high levels of proline under stressed conditions (Figure 3b). Proline is a significant indicator for finding out how tolerant plants are under water-restrictive conditions [71]. Proline protects plants from the hazards of dehydration stress via maintaining osmotic adjustment, maintains membrane integrity, and enhances the antioxidant defense system [72,73]. Proline accumulation in TRIA-treated seedlings exposed to either drought or salt stress has been reported by [31,74].

In the present work, rice exposure to drought stress led to oxidative imbalance as indicated from the marked increase in the percentage of electrolyte leakage (EL) and malondialdehyde (MDA) content (Figure 4), as well as the significant decrease in photosynthetic pigments, which may be a result of its photo-oxidation and degradation under the effect of accumulated free radicals, induced by oxidative stress [75]. In response to TRIA priming, the reverse was true.

Hence, it was reported that a major requirement for plants to tolerate drought stress is the ability to withstand and/or counteract the oxidative imbalance associated with the decrease in available water. TRIA, in this regard, played a crucial role in lessening the hazards of oxidative stress via increasing the content of free amino acids in stressed plants, which among other effects, could mitigate oxidative stress via reducing reactive oxygen species [76]. The increase in total soluble sugars and free amino acids may be attributed to the acceleration of the photosynthesis process via increasing the photosynthetic pigment contents. In this context, the current study showed that TRIA increased the Chl a and Chl b contents compared with the stressed plants. Such an effect of TRIA may be attributed to its role in protecting the chlorophyll from oxidation by increasing the carotenoid content in TRIA-treated plants (Table 3). Carotenoids act as an important antioxidant protecting pigments from the oxidation induced by the stressful condition [77]. Chlorophyll fluorescence is a good indicator of stress tolerance [78], and in the current study, TRIA treatment showed an increment in Fv/Fm values under drought stress conditions, which refers to a higher photochemical efficiency of PSII [59]. It was reported that TRIA has also increased the Fv/Fm values under various abiotic stresses, such as salt and chilling stresses [29,30].

Predicted functions of sugars and amino acids, particularly of those which are hydrophilic, for the improvement of the water status of plants subjected to drought stress include the following: water replacement molecules, when acting as protectants, and stabilizing subcellular structures in drought conditions [79]. Moreover, some of these amino acids and sugars, which have polar groups within their structures, may coat intracellular macromolecules with a cohesive water layer providing preferential hydration to these molecules and, hence, more retention of water under its scarcity [80].

The alleviation of oxidative stress by TRIA in drought-stressed plants could be achieved via reducing membrane injury [81]. Likewise, the present study showed that TRIA notably decreased EL in association with a decrease in the lipid peroxidation product MDA content as compared with drought-stressed plants (Figure 4a,b). In this context, TRIA has been observed to reduce membrane permeability and MDA contents in maize seedlings under salinity stress [32]. Such an effect of TRIA may be attributed to the activation of some antioxidant enzymes that contribute to buffering the excess reactive oxygen species (ROS), which results in alleviating the stress damage effects on plants. It was reported in many studies that TRIA stimulated the activity of some antioxidant enzymes under drought and salinity stresses [31,65,82].

Aquaporins (AQPs) play a crucial role in regulation of water transport through plants; hence, they take part in drought stress tolerance. Plasma membrane intrinsic proteins (PIP) belong to one of the subfamilies of AQPs, and PIPs are further subdivided into two phylogenetic subgroups: PIP1s and PIP2s. In the current study, the increase in water content in TRIA-treated plants was accompanied with the upregulation of *PIP1,1*, *PIP1,2*, *PIP2,4,* and *PIP2,5* genes (Figure 5a–d) in both the shoots and roots of plants, either under normal or drought conditions. In this context, it was reported that TRIA can alleviate the toxic effects of stress by regulating the gene expression [28,32]. Generally, the downregulation of specific *PIP* isoforms leads to a decrease in water permeability of protoplasts, and consequently increases susceptibility to drought and osmotic stress [83,84]. Hence, *PIP* isoform overexpression participates in the increments of root osmotic hydraulic conductivity [85,86]. Moreover, the overexpression of *PIPs* genes in TRIA-treated plants may be attributed to the accumulation of ABA [87,88]. It was reported in many studies that the application of exogenous ABA increased *PIP* gene expression under normal water supply [89,90,91]. In addition, the accumulation of ABA under drought stress plays a crucial role in regulating AQP gene expression [87,88].

## 5. Conclusions

The current results provide molecular and physiological evidence supporting the vital roles of triacontanol in improving the water status in drought-stressed rice seedlings, which may play a beneficial role in horticultural crop management to tolerate climatic fluctuations. The obtained results showed that TRIA alleviated the adverse effects caused by drought stress through molecular and physiological strategies, which contribute to improving the water status. Moreover, TRIA via increasing the content of free amino acids and soluble sugars under drought stress may increase the efficiency of stressed plants to retain water under its scarcity. Such an effect of TRIA was evident by the increase in the RWC and decrease in the MDA content and EL. In addition, TRIA highly induced the expression of aquaporin-related genes (*PIP1,1*, *PIP1,2*, *PIP2,4,* and *PIP2,5*) that might be involved in the regulation of water transport.

## Figures and Tables

**Table 1:** Some physical and chemical properties of soil.

pH	Electrical Conductivity (EC) (dS m^−1^)	Clay (%)	Silt (%)	Sand (%)	Texture
8.2	2.8	53.3	31.4	15.3	Clayey

**Table 2:** The primers used for real-time PCR analysis.

Primer Name	Primer Sequence 5′-3′	Gene Accession Number
PIP1,1 F′	TGCGCAGCCGACGACATG	AK061769
PIP1,1 R	CATACAGTGACTGAGTACTGGATTAC	
PIP1,2 F	CTGTCAAGATGCCAATCCAGAG	AK098849
PIP1,2 R	GAACCGAACTCCAATAGGAGGA	
PIP2,4 F	GAGCTCGTCTGGTGATATCC	AK072632
PIP2,4 R	CATGAAGACAACAGAGGGACAG	
PIP2,5 F	GCTTAAGCCGCAATCAAATGTGC	AK107700
PIP2,5 R	CGATCGAACAATGTCACACTTGC	
OsActin F	CTGGGTTCGCCGGAGATGAT	XM_015774830.2
OsActin R	TGAGATCACGCCCAGCAAGG	

**Table 3:** Effects of TRIA (35 ppm) on photosynthetic pigment contents (µg/g FW) and the maximal photochemical efficiency of the primary photochemistry (Fv/Fm) of the leaves of rice seedlings exposed to drought stress. Data are means of three replicates ± SE.

Treatments	Chl (a)	Chl (b)	Carotenoid	Fv/Fm
Control	0.59 ± 0.0058 b	0.26 ± 0.013 c	0.39 ± 0.00 b	0.78 ± 0.012 c
Control-TRIA	0.79 ± 0.023 a	0.31 ± 0.0135 b	2.4 ± 0.205 a	0.93 ± 0.09 a
Drought	0.29 ± 0.012 d	0.25 ± 0.015 c	0.24 ± 0.012 b	0.04 ± 00.00 d
Drought-TRIA	0.52 ± 0.015 c	0.39 ± 0.006 a	0.5 ± 0.006 b	0.7 ± 0.006 b

Columns with different letters are significantly different at *p* ≤ 0.05.

## Data Availability

The data presented in this study are available in this manuscript.

## Abbreviations

Chl a	chlorophyll a
Chl b	chlorophyll b
EL	electrolyte leakage
Fv/Fm	the maximal photochemical efficiency
ABA	Abscisic acid
AQPs	Aquaporins
*PIP1,1*, *PIP1,2*, *PIP2,4* and *PIP2,5 *	Plasma membrane intrinsic proteins 1,1; 1,2; 2,4; and 2,5 genes
TRIA	triacontanol
RWC	relative water content
